# Letter from Berlin

**Published:** 1890-01

**Authors:** 


### Letter from Berlin.

Professor Brieger on Bacteria and Ptomaines.—Toxines and their Effects.—Cholera Bacilli and Cholera Colors.—Tetanin.—Auto-intoxication.

The powerful chemical actions of microbic organisms can be observed throughout nature. The various fermentative processes, the changes in the soil after tilling, and the transformation of insoluble matters into solubles, and, for plants, assimilizable ones, are all chiefly the work of micro-organisms. The study of the chemical relations of bacteria is consequently of eminent importance for the physician who desires to comprehend the symptomological aspects of diseases of parasitic origin. For it must be admitted that the mere mechanical propagation of microbes and the subsequent abstraction of albumin and oxygen from the blood, do not suffice to explain all phenomena connected with zymotic diseases. Bacteria being living organisms it is clear that they can not exist except by feeding upon their surroundings, and that on the other hand, they must excrete waste materials, which, in this instance, find their way into the circulation. The waste materials of a basic nature are clinically the most important ones, as they are able not only to damage, but also to destroy the human organism. We distinguish between toxines, the more poisonous microbic excretions, and ptomaines, those of a less virulent nature. The number of chemically well-established toxines and ptomaines which we have been enabled to prepare in a pure state is about forty-five, of which Professor Brieger has found nearly thirty himself. This number includes also the ptomaines which cause sickness en masse, such as is caused by deteriorated victuals. In the little clams (mytula edulis), of Wilhelmshafen, which a few years ago caused the death of many persons, Brieger found mytilotoxine, a body which hitherto has been found nowhere else, and which with chloride of gold forms a beautiful crystalline combination. Neurin and methylguanidin, however, which belong to the same order of toxines, can be found in various zymotic affections. The symptoms presented by a person poisoned by these little clams were a peculiar sense of constriction in the throat, mouth, and lips, burning in the extremities, numbness in the head, and a sensation as if the limbs wanted to fly upward. Every object lifted appeared of a wonderful lightness and seemingly tended to rise spontaneously. Suddenly the pupils dilated and great physical excitation set in, the patients ran about nervously until suddenly exhaustion and collapse supervened. Death was preceded by a profound fall of the temperature of the skin, violent vomiting and total loss of power. Five or six clams sufficed to cause severe attacks in adults. Some interesting experiments on animals were made with the water in which the clams had lived, and which, of course, after having been boiled did not contain any living bacteria, but only their toxine. The animals after taking this water soon began to move their heads to and fro, tried to escape under great excitement and dyspnœa, but did not get far, their legs suddenly becoming paralyzed. The muscles refused to work, and the animals sank down in utter exhaustion and perished during convulsions. Even after death the activity of the toxine does not cease, but being no longer impeded by the vital resistance of the cells of the organism it acts on the contrary, all the more violently and hastens the dissolution of the body. New toxines arise on the seventh day after death, particularly mydaleine, which even in small doses causes the death of animals with symptoms of violent diarrhœa, vomiting, and gastro-intestinal inflammation.

Of more general clinical interest are such toxines and ptomaines as arise from the actual virulence of certain bacteria. Staphylococci and streptococci, the microbic causes of pyemia and septicemia, present different chemical relations and consequently different manifestations of their destructiveness. From cultures of Koch's typhus bacillus we can prepare a toxine called typhotoxine, which injected into guinea pigs causes paralysis of the voluntary muscles and increase of the salivary and intestinal secretions. The chemical virulence of Koch's cholera bacillus is particularly terrible, it generates, together with various special toxines, tetra- and penta-methylen-diamin and methylguanidin.

This chemical action of the cholera bacillus explains the local intestinal irritation, the violent diarrhœa, the loss of the coagulability, and the varnish color of the blood, the frigidity and muscular cramps, and even the peculiar odor of the expired air of cholera patients. It is peculiar to the cholera bacillus that it abstracts from the soil on which it is artificially cultured certain substances which, after addition of concentrated sulphuric acid, produce a beautiful blue or burgundy-red fluorescent color. The colors thus produced are known by the name of cholera blue, or cholera red, and are genuine dyes.

From an arm just amputated from a tetanus patient we can prepare tetanin, a most virulent poison. Tetanin is a toxine excreted by Nicolaier's tetanus bacillus which originally exists in the soil, but, as Rosenbach has proved, can emigrate to the human organism. Besides this toxine, the tetanus bacillus is capable of producing four other highly virulent toxines. Introduced into the animal organism the tetanus bacillus produces violent muscular spasms, cramps, and lockjaw, with its usually fatal results. It is singular, that the introduction of toxines into the organism deprive the latter of its power to resist the entrance of dangerous or specific microbes. This, of course, constitutes a highly dangerous property of toxines. Hueppe has proved that cholera bacilli which could not enter the healthy organism find their way into the latter readily and in great numbers, if their toxines have been previously admitted. The experiments of Ehrlich and Brieger have also proven that the specific poison of typhus renders the system susceptible to the admission of the microbes of malignant œdema, which are harmless to a healthy organism.

Further studies on the chemico-bacterial poisons will probably decide whether the hitherto only hypothetical doctrine of the auto-intoxication of the human body can be regarded as a scientific fact. Much more desirable, however—and this is within reach of probability—would be the capacity to afford to the system immunity against the immigration of pathogenic bacteria by means of inoculation with their toxines. For as bacterial diseases are principally intoxications, the degree of susceptibility to infections is proportionate to the greater or lesser susceptibility of the individual to the poisons. Trials with inoculations of devitalized cultures of pathogenic microbes have been successful in chicken cholera, anthrax, typhus fever, etc. But there can be no definite results in this regard without the employment of the toxine prepared pure and chemically. Chemistry and internal medicine will have to join hands in order to attain results which shall benefit the suffering community at large.

